# Embracing uncertainty: a core competency for the global orthopaedic surgeon

**DOI:** 10.1051/sicotj/2026007

**Published:** 2026-05-26

**Authors:** Yogesh S. Salphale

**Affiliations:** Consulting Orthopaedic & Trauma Surgeon, Shushrusha Multispecialty Hospital Chandrapur India

**Keywords:** Uncertainty, Global orthopaedics, Surgical decision-making, Resource-constrained settings, Professional competence, Ethical practice

## Abstract

Orthopaedic surgeons across the world work within uncertainty. Decisions are often shaped not only by fracture patterns and imaging, but also by delayed presentation, limited resources, variable rehabilitation access, financial constraints, and differing patient expectations. In many global settings, especially resource-constrained environments, uncertainty is not an occasional inconvenience but a constant feature of clinical practice. This editorial argues that the capacity to recognize, tolerate, and respond constructively to uncertainty should be regarded as a core professional competency for the global orthopaedic surgeon. Rather than viewing uncertainty as a weakness or failure of knowledge, surgeons should approach it as a space that demands judgment, adaptability, humility, and ethical clarity. Training and professional discourse in orthopaedics should therefore move beyond technical mastery alone and acknowledge uncertainty management as central to sound decision-making, context sensitive care, and responsible surgical leadership.

Uncertainty is often perceived as an inconvenience – yet across the global orthopaedic landscape, it is a defining feature of practice. Surgeons routinely confront situations where available information is incomplete, diagnostic clarity is delayed, and decisions must be made before the full picture is known. Recognising this reality is essential if the profession is to cultivate a generation of surgeons prepared for modern complexity [1].

Even in well-resourced hospitals, uncertainty persists. Technology improves visibility but does not eliminate the inherent biological variability that shapes outcomes. As orthopaedic care globalises, surgeons increasingly encounter unfamiliar clinical contexts – different comorbidities, social conditions, and follow-up environments – each adding layers of unpredictability to decision-making [2].

Training often emphasises correctness – identifying classifications, selecting implants, and replicating techniques – yet much of orthopaedic surgery occurs in situations where no single option is unquestionably superior. A more explicit engagement with uncertainty during training would strengthen clinical judgement and reduce the risks associated with overconfidence [1, 3].

Communication becomes the most reliable tool when certainty fades. Patients rarely demand absolute clarity; they seek honesty, transparency, and shared decision-making. Surgeons who articulate what is known, what remains uncertain, and how they intend to proceed foster trust and improve adherence to treatment recommendations [4].

Uncertainty is magnified in health systems where resources are constrained. The goals of Global Surgery 2030 highlight the need to strengthen decision-making capacity and surgical systems in environments where unpredictability is intrinsic rather than exceptional [5].

Innovation frequently originates not from abundance but from necessity. Context-appropriate solutions – whether simplified workflows or locally feasible fixation strategies – demonstrate that uncertainty can stimulate problem-solving rather than hinder progress. Adaptive resilience is seen when theatre teams use manual suctions and portable headlights in places where power failures are frequent. For institutes lacking antibiotic spacers, hand-mixed PMMA beads using available antibiotics help in the effective local control of infection.

A professional culture equating confidence with certainty may inadvertently discourage trainees from acknowledging doubt. Normalising thoughtful discussions about uncertainty during rounds, trauma meetings, and morbidity reviews cultivates humility and supports safer decision-making.

## Conclusion

Uncertainty is not a barrier to surgical excellence. It deepens judgment, sharpens situational awareness, and supports responsible innovation. Embracing uncertainty as a teachable and discussable component of orthopaedic care will prepare future surgeons to lead confidently in diverse and unpredictable clinical environments.

## Data Availability

No datasets were generated or analyzed during the current study.
